# An Evaluation of the Design and Usability of a Novel Robotic Bilateral Arm Rehabilitation Device for Patients with Stroke

**DOI:** 10.3389/fnbot.2017.00036

**Published:** 2017-07-28

**Authors:** Yu-Cheng Pei, Jean-Lon Chen, Alice M. K. Wong, Kevin C. Tseng

**Affiliations:** ^1^Department of Physical Medicine and Rehabilitation, Chang Gung Memorial Hospital at Taoyuan, Taoyuan, Taiwan; ^2^School of Medicine, Chang Gung University, Taoyuan, Taiwan; ^3^Healthy Aging Research Center, Chang Gung University, Taoyuan, Taiwan; ^4^Center for Vascularized Composite Allotransplantation, Chang Gung Memorial Hospital at Linkou, Taoyuan, Taiwan; ^5^Product Design and Development Laboratory, Department of Industrial Design, College of Management, Chang Gung University, Taoyuan, Taiwan; ^6^Department of Design, National Taiwan Normal University, Taipei City, Taiwan

**Keywords:** upper limb rehabilitation, stroke, rehabilitation device, usability, bilateral movement therapy

## Abstract

**Study design:**

Case series.

**Evidence level:**

IV (case series).

**Introduction:**

Robot-assisted therapy for upper limb rehabilitation is an emerging research topic and its design process must integrate engineering, neurological pathophysiology, and clinical needs.

**Purpose of the study:**

This study developed/evaluated the usefulness of a novel rehabilitation device, the *MirrorPath*, designed for the upper limb rehabilitation of patients with hemiplegic stroke.

**Methods:**

The process follows Tseng’s methodology for innovative product design and development, namely two stages, device development and usability assessment. During the development process, the design was guided by patients’ rehabilitation needs as defined by patients and their therapists. The design applied synchronic movement of the bilateral upper limbs, an approach that is compatible with the bilateral movement therapy and proprioceptive neuromuscular facilitation theories. *MirrorPath* consists of a robotic device that guides upper limb movement linked to a control module containing software controlling the robotic movement.

**Results:**

Five healthy subjects were recruited in the pretest, and 4 patients, 4 caregivers, and 4 therapists were recruited in the formal test for usability. All recruited subjects were allocated to the test group, completed the evaluation, and their data were all analyzed. The total system usability scale score obtained from the patients, caregivers, and therapists was 71.8 ± 11.9, indicating a high level of usability and product acceptance.

**Discussion and conclusion:**

Following a standard development process, we could yield a design that meets clinical needs. This low-cost device provides a feasible platform for carrying out robot-assisted bilateral movement therapy of patients with hemiplegic stroke.

**Clinical Trial Registration:**

identifier NCT02698605.

## Introduction

The World Health Organization (WHO) has reported that stroke is the third leading cause of death in developed countries and involves approximately 15 million stoke events annually. One-third of stroke patients die and a further one-third of events results in permanent disability. Depending on the location of the brain insult, stroke can lead to a wide range of functional impairments (Mackay et al., 2004); these include language, cognition, sensation, and motor functions. Motor impairment impacts the patient’s ability to perform activities of daily living. For the majority of patients, recovery of motor function involving an upper limb is slower than that of lower limb (Feys et al., 1998). Indeed, most activities of daily living rely the functioning of the upper limb, thus emphasizing the need for effective upper limb rehabilitation.

With an attempt to enhance the effectiveness of upper limb rehabilitation among stroke patients, a series of rehabilitation techniques have been developed and refined in recent decades; these include task-oriented motor training, constraint-induced movement therapy, mirror therapy, and bilateral movement training. Each of these methods has a number of theoretical advocates and each has been shown to be effective clinically. For instance, bilateral movement therapy, which involves coordinated movement of the bilateral upper limbs, has been shown to enhance upper limb recovery and coordination between the hands. Stoykov et al. (2009) found that bilateral arm training is more effective than unilateral training when restoring proximal upper limb function because it seems to improve the functional linkages between the bilateral hemispheres.

Even after receiving a full course of conventional rehabilitation, 60% of stroke patients still have difficulties when using their affected upper limb (Kwakkel et al., 1999). As a result, it has become the upmost importance to develop novel rehabilitation strategies that are able to help patients reach a higher level of recovery. One such approach is robot-assisted rehabilitation, which incorporates robotic technologies into the rehabilitation processes. Several well-known robot-assisted movement therapies for the upper limb has been implemented clinically, including MIT-Manus (Krebs et al., 1998), Bi-Manu-Track (Hesse et al., 2003), BATRAC (Cauraugh et al., 2010), and MIME (Burgar et al., 2000), each of which follows different movement therapy theories. Regarding the body parts that are mainly involved in therapy, Bi-Manu-Track focuses on the bilateral forearms and wrists, while BATRAC and MIME focus on the shoulder and elbow of the affected limb. Regarding the movement dimension, BATRAC involves movement in one-dimension, while MIME allows three-dimensional movement. In fact, the higher the degrees of freedom adopted during the movement therapy, the more complex is the design of the robotic device. As a result, it has become important to come up with a feasible design that fulfills the patient’s rehabilitation needs while avoiding the high costs that can be associated with instrument acquirement and maintenance. Furthermore, the effectiveness of the system needs to be comparable to that provided by conventional therapies so that a motivation to pursue this therapeutic option can be established (Kwakkel et al., 2008; Lo et al., 2010).

As an approach to the development of mechanical rehabilitation devices for hemiplegic upper limbs, Timmermans et al. (2009) proposed that three design domains are required; these were the therapy techniques used, the motivation of the patient, and resulting performance rewards. An online survey of physical therapists, 233 in total, indicated that a preferred upper limb robotic device needs to accommodate different hand movements, to be able to be used while in a seated position, to be able to provide the user with feedback, to focus on the restoration of activities of daily living, to able to be used at home, to have adjustable resistance levels and to cost less than US$6,000 (Lu et al., 2011).

In terms of usability, the interaction between the user and the machine tends to be overlooked during the development stage. Although a variety of upper limb rehabilitation machines have been proposed, only a few have been commercialized. This low market acceptance can be attributed to the high cost of these devices, safety concerns, and poor usability (Lee et al., 2005). To this end, the aim of this study was to design a bilateral upper limb rehabilitation device called *MirrorPath* for the rehabilitation of stroke patients that follows the theories of bilateral movement therapy and proprioceptive neuromuscular facilitation (PNF). These two theories were initially developed by Knott and Kabat and have been shown to have a positive effect on the range of active and passive motions needed by stroke patients (Sharman et al., 2006). Our device will guide the patient’s upper limbs, each of which moves along a diagonal motion path on the horizontal plane. The position and velocity of motion of the bilateral limbs are perfectly mirrored across the midline on the table. Finally, usability testing was conducted on the completed prototype.

## Materials and Methods

This study adopts Tseng’s methodology (Tseng, 2013) for innovative product design and development. This consists of two stages: (1) the device development and (2) usability assessment stages.

### Stage 1: Device Development

This study began with observations at the Rehabilitation Center at Chang Gung Memorial Hospital, Taoyuan, Taiwan, targeting rehabilitation activities, and carrying out interviews of stroke patients and their therapists; this is because device development must be prefaced by an understanding of the users’ needs. In order to allow patient privacy, non-participant observation techniques were used, with a focus on manual pen-and-paper observations. In addition, interviews were conducted with an occupational therapist and a physical therapist. To better understand patients who were potential device users, personal and situational analyses were used to explore potential product usage issues and to integrate the observation and interview results into the design process.

It was concluded that the ability to perform the rehabilitation movements needed to be the primary design consideration. The primary design consideration then determines future design factors, such as the appropriate movement mechanisms to be used, the choice of motors, and various ergonomic considerations. In this study, we have emphasized the rehabilitation of the proximal upper limbs and, therefore, any systems need to focus on shoulder and elbow movements. The motor that drives the patient’s arm movement, therefore, needs to exceed the maximal mechanical loading predicted by the weight and resistance of the upper limb and the friction force of the apparatus. According to the 2005–2008, National Nutrition and Health Survey, the average weight of adult males and females in Taiwan for this period were 69.6 and 58.0 kg, respectively. The weight of an individual’s arm was then estimated as a proportion of total mass using gamma-ray measurements, which arrived at the average weights of single arms of 3.7 kg (36 N) and 2.9 kg (28 N) for adult males and females, respectively. In that, an additional safety factor was added to give a total estimated mechanical load of 54 N. The device’s dimensions were designed to accommodate up to the 95th percentile of the height of female adults aged 45–65 years old. Specifically, in this case, the 95th percentile of female adult was a shoulder-to-radial-styloid distance of 439 mm.

### Stage 2: Usability Assessment

This study protocol was specifically approved by the Institutional Review Board of Chang Gung Medical Foundation with ID 101-5038A3. The study has been registered in https://ClinicalTrials.gov with registration number NCT02698605. The usability assessment focused on the actual use of the proposed rehabilitation system in order to clarify the issues that users might face during actual system operation, which would provide a reference for subsequent system improvement. Pretesting and formal testing were conducted at Chang Gung Memorial Hospital, Taoyuan, Taiwan, a tertiary hospital that provide comprehensive neurorehabilitation. The pretesting was recruited through self-selection and formal testing through referral. The pretesting was conducted on five healthy participants, while the formal testing was conducted on 12 individuals (four stroke patients, four caregivers, and four therapists). The sample size was chosen according to the suggestion of Lewis and Sauro (2009), as a subject number of 12 was considered adequate for usability analyses. The healthy participants were aged 20–70 years old and had no physical disabilities; the stroke patients had normal cognitive and language skills, a stable stroke status, no fractures affecting the upper limbs during the previous 3 months, and minimal or no upper limb spasticity, namely a Modified Ashworth Scale of 0 or 1. The occupational therapists had work experience in the hospital for more than 1 year.

**Figure 1:**
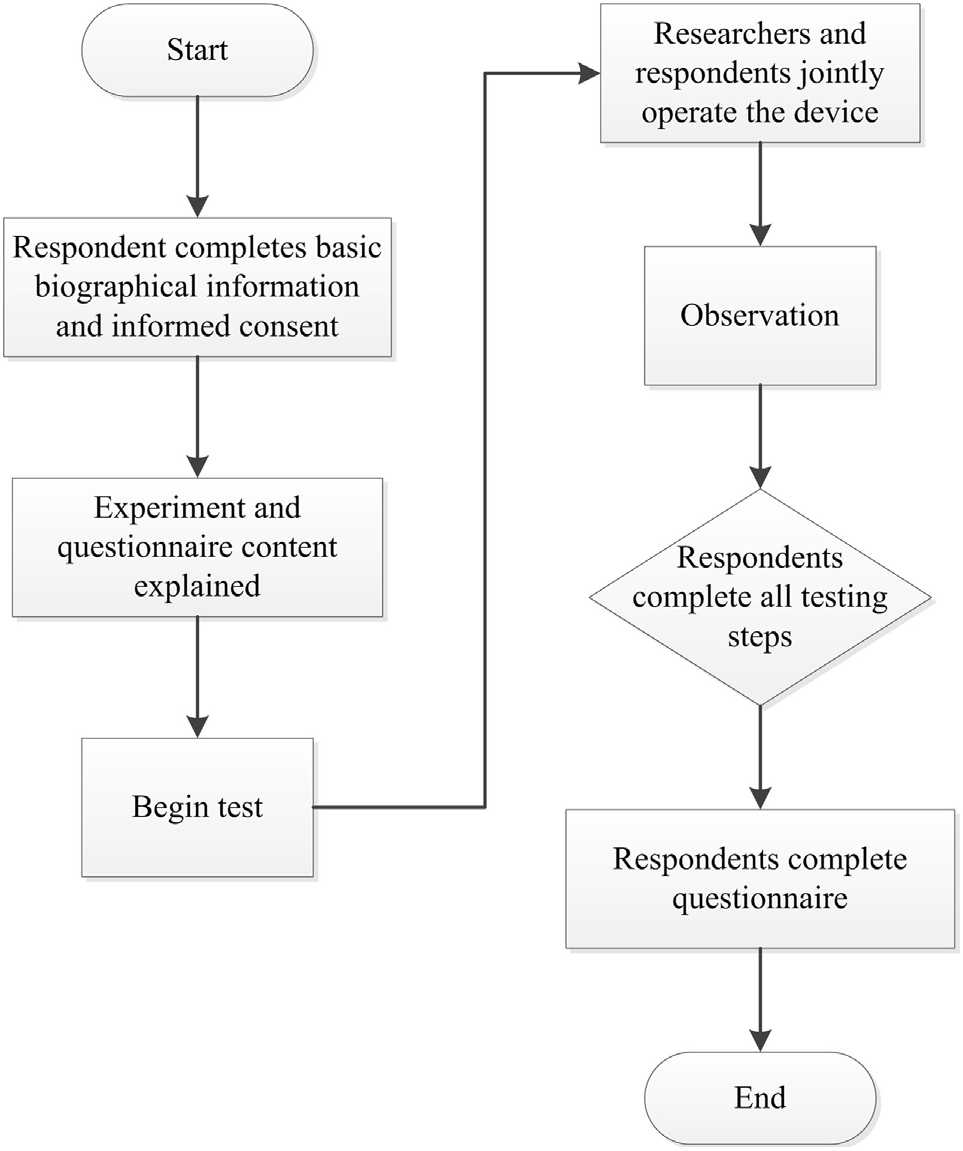
The flow of a usability test.

This study was a one-arm study and all subjects used the device and received usability test. The study was conducted by research assistants. Each subject received one assessment that lasted 30 min. The subjects were not paid for participation. In that, no randomization or masking was performed. Prior to the experiment part of the study, the subjects provided basic biographical information. We then explained the experimental process and demonstrated the operation of the device. For usability assessment, the subject operated the device under the instruction of the researchers, during which problems were identified and any questions asked by the participants recorded. The primary outcome was the system usability scale (SUS) questionnaire and the secondary outcomes were observational recording forms and open questions the subject reported during the assessment. Following device operation, the subjects completed the SUS questionnaire that collected subjective evaluations and recommendations regarding the device. The experimental instruments used in this part of the study consisted of the rehabilitation device, video cameras, still image cameras, digital voice recorders, questionnaires, and observational recording forms (Figure 1).

The SUS was developed by Brooke (1996) as a system usability tool, which has been widely used in the evaluation of a range of systems (Brooke, 2013). The results of the SUS questionnaire were analyzed using SPSS. The split-half method was used for the reliability analysis and the one-sample *t*-test was used for statistical comparisons.

## Results

### Stage 1: Device Development

#### Observations and Interviews

The observation of patients with a need for upper limb rehabilitation showed the following clinical problems: (1) the patients had insufficient upper limb strength, (2) the patients had insufficient motivation to carry out autonomous rehabilitation, (3) the patients needed resistance adjustment to be available as part of the rehabilitation device, (4) the patients were insufficiently aware of their own rehabilitation status, and (5) the available therapist-to-patient ratio is low and this would result in a situation where rehabilitation is unsupervised from time to time.

The results led to the following clinical recommendations: (1) due to the lack of therapist manpower, therapists may need to supervise more than one patient at a time, (2) additional approaches are needed to encourage patients to achieve rehabilitation goals, (3) rehabilitation goal setting is highly reliant on patient intention including the extent of the exercise carried out, their learning status, their willingness to engage in rehabilitation, and (4) the design of the arm rehabilitation activities needs to be largely determined by the materials and equipment available to the therapist. The recommendations for the rehabilitation operation are that: (1) the use of fixed paths will be able to help the patient complete the rehabilitation exercise more smoothly and (2) the device needs to be suitable for patients with Brunnstrom stages 1–4 (Brunnstrom, 1966).

#### Scenario Analysis

Scenario analysis was based on on-site observations and the literature, and these were used to establish four stroke patient archetypes (see Table 1). The four stroke patient archetypes were constructed to allow the scenario analysis to be carried out based on the results obtained during the on-site observations. These representative archetypes ought to be able to account for the behavior of four completely different patient types. We then used a situational narrative approach to explore the usability problems with respect to the aforementioned stroke archetypes and to propose potential solutions.

**Table 1:** Role and situational narrative descriptions with respect to the four stroke patient archetypes.

Name	Gender	Age	Personality	Educational attainment	Stroke status	Muscle power	Rehabilitation status	Narrative	Issues	Potential solutions
Ta-Chun Chang	Male	46	Mild-tempered, detail-oriented	Master degree	Right UL Brunnstrom stage 3	Right side: proximal 3, distal 2; left side: 5	Strong motivation	The patient is tall. If the device height is fixed, the patient will be positioned on an uncomfortable posture	Device height must be adjustable to match the height of each user	Adjustable device and chair height

Lucy Wang	Female	75	Unassertive	Junior high school	Right UL Brunnstrom stage 2	Right side: proximal 2, distal 1; left side: 5	Poor frustration tolerance	The patient needs constant attention from the therapist. Her concern is that she cannot stop the machine by herself if any safety issue occurs	Therapists are not always close by, which raises safety concerns	Emergency cut-off switch Voice-activated control

Helen Chen	Male	55	Generous	Junior high school	Left UL Brunnstrom stage 3, motor aphasia	Left side: proximal 3, distal 2; right side: 5	Impatience, easy tantrum	The patient hopes the family would be able to participate in the rehabilitation activities. Poor motivation was observed whenever the family was not around	Unstable motivation has resulted in poor progression during rehabilitation	Time control 1-on-1 work with the therapist

Ta-Tung Lee	Male	83	Macho, patriotic, bad temper	Elementary school	Right UL Brunnstrom stage 4	Right side: proximal 4, distal 3; left side: 5	Strong motivation, but little progress in functional recovery	Despite prolonged rehabilitation for months, his slow functional recovery has distressed the patient	Timely feedback on rehabilitation performance is needed	Documented rehabilitation record Game mode for instantaneous feedback

*UL, upper limb*.

##### Design Conditions

Most rehabilitation devices, such as push–pull boxes, pulleys/tackle, and stacking require autonomic exertion by the patient. This study, however, is primarily concerned with stroke patients who have limited functionality in term of this level of autonomy. We proposed a rehabilitation device that not only maintains the motor activity and the range of motion of the upper limb but also induces better scapula motion than BATRAC-like devices. Observations, interviews, the Persona method, and situational narratives were used to explore potential usage problems. The results raised the following design specifications that would be required to fulfill the patients’ rehabilitation needs: (1) an auxiliary arm support, (2) an auxiliary handle, (3) active machine movement, (4) bilateral mirrored action, (5) a fixed path, and (6) an emergency cutoff.

#### Proposed Design

**Figure 2:**
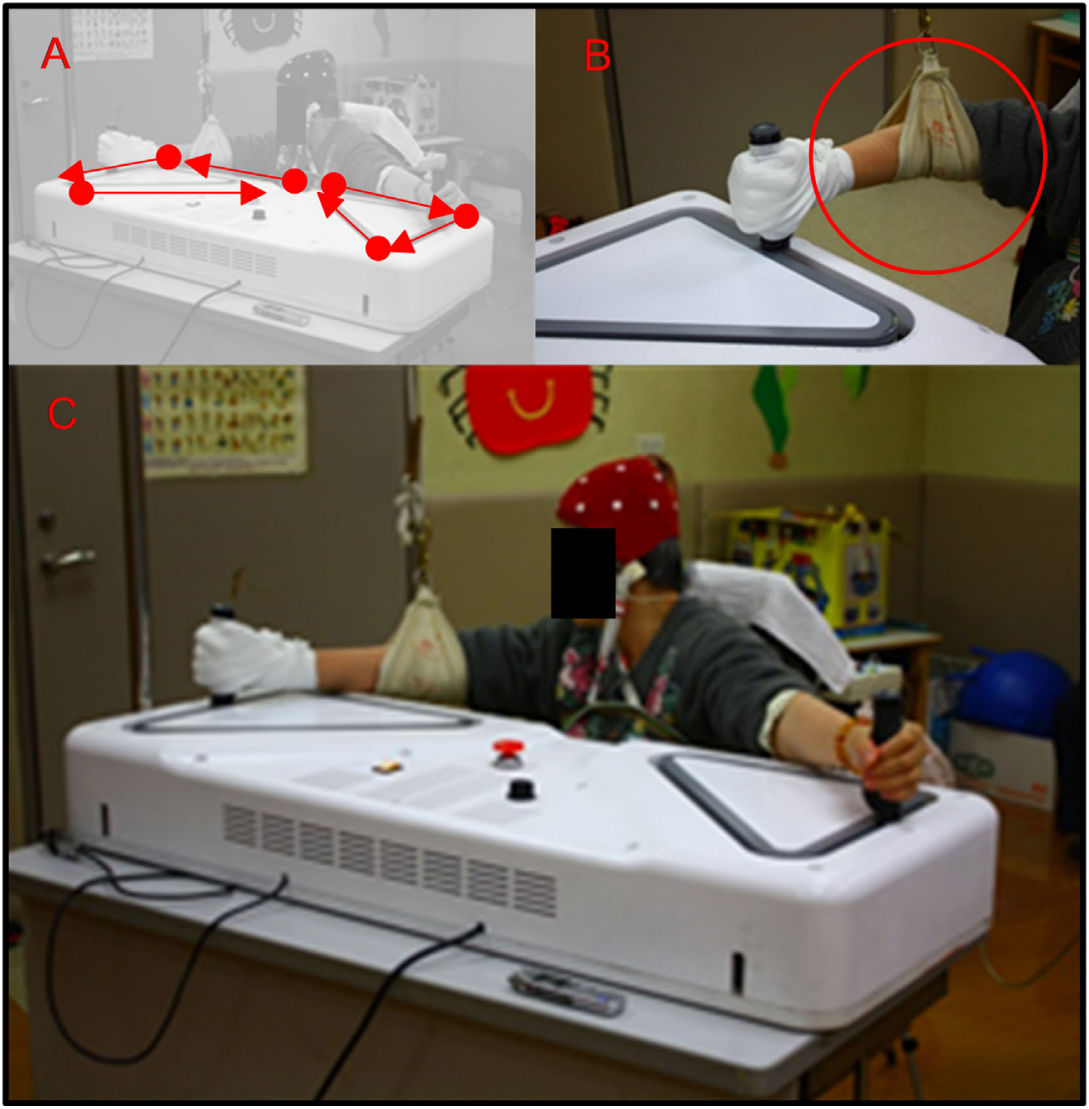
**(A)** A patient performed bilateral diagonal movements using the device; **(B)** due to weakness of right upper limb, the patient’s grip was assisted with an elastic bandage, and the patient’s elbow was support by a sling; **(C)** the application scenario.

Based on the design specifications described above, we developed a prototype (Figure 2) that was 1,180 mm × 440 mm × 300 mm in size and had a shell constructed of acrylonitrile butadiene styrene (ABS). The control module featured an on/off switch, a knob for adjusting the speed, and an emergency cut off switch.

The patient’s hands grip each of the handles. An electric bandage was applied to assist grip when the hand on the affected side was unstable with respect to griping the handle. The movement of both arms was driven by a servo motor operating at 6,500 rpm, with a force of 0.28 N. The force was then transmitted *via* two gear ratios (1:5 and 1:3). As the device is decelerated by these gears, the torque is increased to 12.6 N, which corresponds to an output force of 420 N. A gear-pulley-belt system is used to drive the handles, because rails cannot be used to form a triangular track (Figures 2A–C). Each upper limb was moved along a triangular pathway on the horizontal plane and the movements of the two hands are perfectly synchronized, yielding perfectly mirrored motion of both upper limbs. Specifically, the right and left upper limbs are guided to move clockwise and counterclockwise, respectively. The speed of motion is adjustable and can be set to range from 20 to 300 mm/s. During the following assessment periods, the speed was fixed at 20 mm/s.

### Stage 2: Prototype Usability Assessment

#### The Pretest

**Figure 3:**
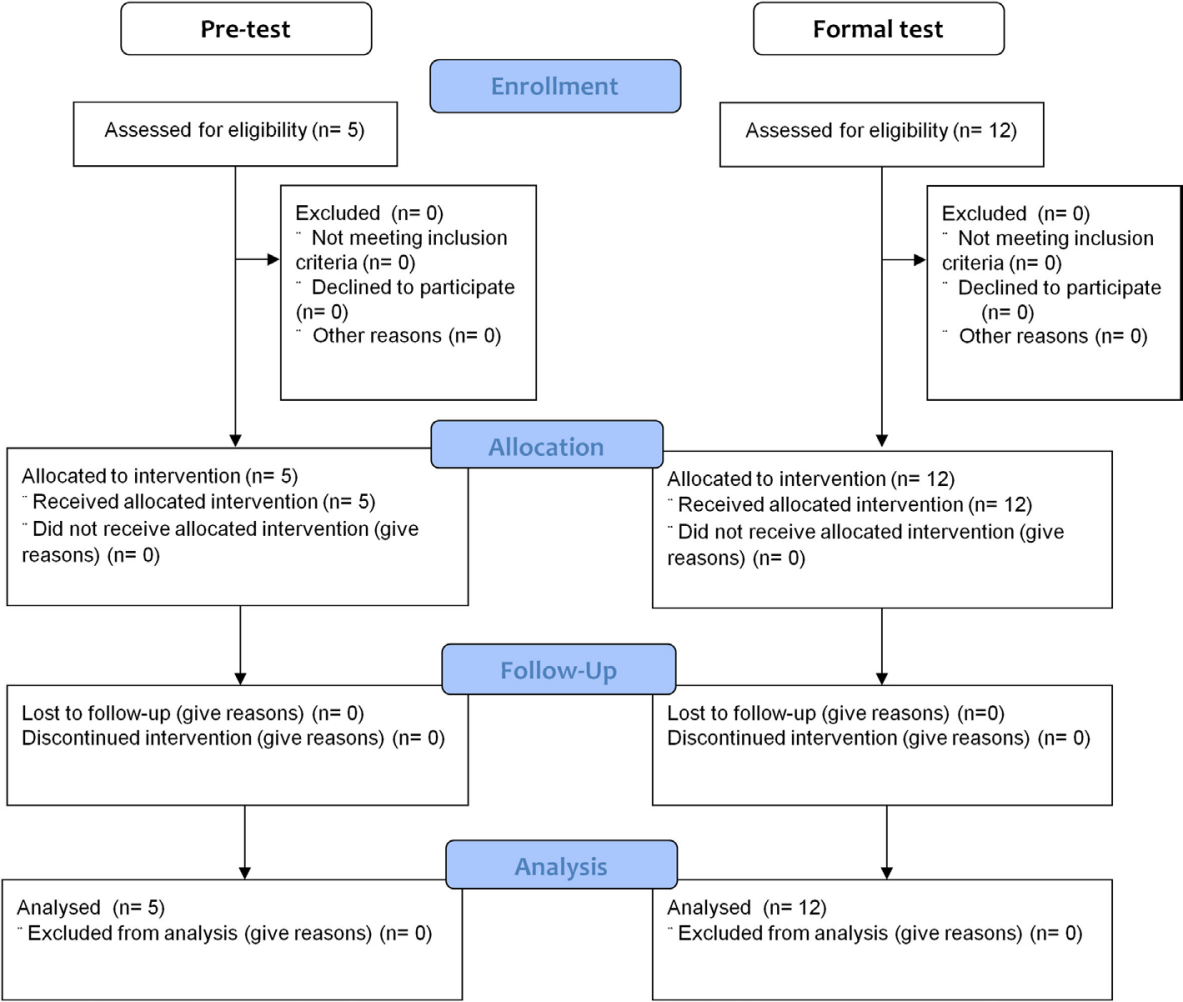
The flowchart for subject enrollment, assignment, allocation, follow-up, and analysis for the pretest and formal test.

Five subjects (one male and four females) participated in the pretest during the recruitment period from 01-08-2013 to 31-01-2014 (Figure 3). The subjects were healthy individuals with ages ranging from 23 to 41 years; they had educational attainment levels ranging from high school to postgraduate studies. None had any previous experience using rehabilitation equipment.

The heights of the chair and the table were adjusted to match the height of the individual subjects. During the operation of the device, we observed that the handle rotates during operation, which may cause discomfort to some subjects who may seek to continuously correct their grip on the handle. Later, we asked participants to grip the handle tightly and they were not to allow to constantly adjust their grip.

During the pretest, the researchers adjusted the device speed three times. Subject 1 indicated that she felt more comfortable at higher speeds, while the others did not express a preference. Generally speaking, subjects felt that the activity was more tedious at slower speeds, but slower speeds are theoretically safer for stroke patients. The therapists suggested that the speed along the hypotenuse part of the movement track should be increased, allowing the subjects to stop in certain positions, and thus spending more time in a stretched positions. Another suggestion was to allow for the direction of the path to be reversed.

The SUS questionnaire score for the subjects ranged from 67.5 to 100, with an average score of 83.5 ± 13.1, indicating a high degree of acceptance (between “good” and “excellent” on the overall usability scale). Categorizing the SUS questionnaire items in relation to usability (Q1, Q2, Q3, Q5, Q6, Q7, Q8, Q9) and learning (Q4, Q10) domains, this showed an average usability score of 85.0 ± 14.2 and an average learning score of 77.5 ± 9.7. This indicates that the device usability domain is more effective than its learning counterpart (see Table 2). Moreover, reliability analysis produces a Cronbach’s α value of 0.96, indicating a very high degree of internal consistency (Cronbach, 1951).

**Table 2:** Total system usability scale (SUS), usability, and learning scores obtained in the pretest.

Item	Total SUS score	Usability score	Learning score
Score	83.5 ± 13.1	85.0 ± 14.2	77.5 ± 9.7

#### The Formal Test

As shown in Table 3, during the recruitment period from 01-02-2014 to 31-01-2016, 12 subjects (five males, seven females), with their age ranging from 25 to 60 years, and their educational attainment levels ranging from elementary school to graduate level, were recruited for the formal test (Figure 3). Two of the subjects had previous experience using robotic rehabilitation equipment systems. Table 4 summarizes the issues raised by the subjects. In total, four issues were raised and the following solutions were created to solve these issues. First, the hand on the paralyzed side was often unable to maintain a grip on the handle. To accommodate this, we applied an elastic bandage that provided additional hand grip support. Second, the height of the instrument was too high for most subjects. To solve this difficulty, we adjusted the chair and table to avoid the problem. Third, the subject’s trunk was forced to bend forward when the motion reached to most forward corner, a problem that was caused by incorrect positioning and the distance between the instrument and subject along the anterior–posterior axis together with inadequate trunk support. As a result, we repositioned the instrument taking in to consideration the subject’s upper limb length. Finally, the belt that guides the movement was quite loose causing the handle to wiggle intermittently. In this case, the tension of the belt was adjusted to minimize this problem.

**Table 3:** Biographical data of the subjects participating the formal test.

Subject number	Gender	Background	Age	Educational attainment^a^	Experience using robotic rehabilitation equipment
01	F	Patient	60	3	No
02	M	Therapist	28	4	No
03	F	Therapist	27	5	No
04	F	Therapist	36	4	No
05	F	Caregiver	25	4	No
06	M	Patient	45	2	Yes
07	F	Caregiver	34	4	No
08	M	Patient	45	4	Yes
09	F	Caregiver	32	1	No
10	M	Patient	49	3	No
11	F	Caregiver	56	2	No
12	m	Therapist	56	4	No

*M, male; F, female*.

*^a^1—elementary school; 2—junior high school; 3—senior high school; 4—university; 5—graduate school*.

**Table 4:** Issues raised by the subjects in the formal test.

Item	Solution
Handle rotates during operation	Application of an elastic bandage to provide additional position support
The machine is positioned too high for most subjects	Adjusted the chair and table to avoid this problem
When the motion reaches the top corner of the triangle, the subject is forced to lean forward to complete the action, and then gradually recover	Re-position the instrument taking in to consideration the subject’s upper limb length
The left side belt is loose, causing the handle to wiggle laterally	The tension of the belt was adjusted to minimize this problem

Table 5 shows that an average SUS score of 71.8 ± 11.9 was obtained during the formal test. As was the case previously, the total usability score (73.7 ± 12.3) was higher than the learning score (64.6 ± 7.8). The SUS scores for each subject, with scores that ranged from 42.5 to 92.5, indicate usability between “Fair” and “Good.” The level of acceptance is high, indicating that the patients and therapists were generally satisfied with the performance of the device.

**Table 5:** Total system usability scale (SUS), usability, and learning scores obtained in the formal test.

Item	SUS score	Usability score	Learning score
Score	71.8 ± 11.9	73.7 ± 12.3	64.6 ± 7.8

The average score for each item ranges from 3.17 to 4.33 (Table 6). Items Q2, Q4, Q8, and Q10 were negative questions, and thus their scores are recoded before calculation, using the equation:

(1) Recoded score=5−original score

**Table 6:** Score for each system usability scale item.

Item	Content	Score
Q1	I think I would like to use the stroke rehabilitation device often	3.67 ± 0.78
Q2	I think the stroke rehabilitation device is difficult to use	4.00 ± 0.43^a^
Q3	I think the stroke rehabilitation device is easy to use	4.33 ± 0.49
Q4	I required technical assistance to use the stroke rehabilitation device	3.17 ± 1.11^a^
Q5	I think the functionalities of the stroke rehabilitation device are well integrated	3.58 ± 1.08
Q6	I think the functionality of the stroke rehabilitation device are not organized	3.58 ± 0.90^a^
Q7	I think most users will be learn to use the stroke rehabilitation device quickly	4.08 ± 0.79
Q8	I think most of users will have difficulties learning to use the stroke rehabilitation device	4.25 ± 0.45^a^
Q9	I am confident when using the stroke rehabilitation device	4.08 ± 0.79
Q10	I may need to learn more background information before I am able to use the stroke rehabilitation device	4.00 ± 0.85^a^

*^a^Recoded by Eq. 1*.

After the recoding, item Q3 (Question “I think this new stroke rehabilitation system is easy to use”) had the highest score, while item Q4 (Question “I required technical assistance to use this new stroke rehabilitation system”) had the lowest score.

Reliability analysis shows a Cronbach’s α value of 0.803 for the SUS scores, again indicating good internal consistency. We further applied the single-sample *t*-test to compare our score to the average SUS score obtained by Sauro (2013) who used a population of 500 different products that have been sold in the market (a score of 68) and found no significant difference [*t*(1) = 1.13, *p* = 0.282]. The results indicate that the usability of our design is not statistically different from those of most market products.

## Discussion

The present study demonstrates the development process of a novel rehabilitation device for stroke patients, which uses the Tseng’s development method (Tseng, 2013). The development process involved the active participation of stroke patients and their therapists, ensuring that there was an understanding of the patients’ needs so that that the product was approached from the point of view of the actual usage conditions during the early development stages. Indeed, even though a literature review and clinical observation can provide insight into basic product design concepts, the developer may still suffer from insufficient understanding of the dynamic interaction between the patient’s physical movement limitations and the robotic device. In this context, the observation stage was able to reveal potential problems that allowed adjustments to be made to design fundamentals. Furthermore, interviews with the therapists, patients, and caregivers further validated the clinical usability of the product. Finally, the creation of subject archetypes and the situational use of narrative methods helped to raise other potential issues and various design modifications were incorporated into the concept sketches and mock-up, allowing inclusion in the engineering specifications.

### Strengths

The significance of this study is that it provides development insight and subsequent usability validation for robotic-assistance rehabilitation devices targeting patients with neurological disorders. The SUS usability questionnaire shows good internal consistency. One important finding is that the pretest SUS score is relatively higher than the formal test core, a difference that might be accounted for by the fact that the pretest was applied to healthy subjects, while the formal test used the actual target subject group, including patients, therapists, and caregivers. Another finding is that usability scores are higher than learning scores, indicating that, if an effective learning method can been applied, then minor usability problems can be overcome. Finally, the usability score was comparable to those obtained from products already on the market, indicating that the rehabilitation device has reach considerable utility even though several minor issues still need to be addressed before it can be applied during advanced clinical research.

During the experimental process, the patients provided positive feedback and indicated they would be willing to use the device for rehabilitation. It is worth noting that the three therapists offered usability scores of 62.5, 72.5, and 42.5. The therapists have occupational knowledge and this gives them particular insights into the actual rehabilitation status of patients and future research should target in-depth interviews with therapists in order to solicit suggestions for improvements to the device. Patients with experience using motorized rehabilitation equipment gave scores of 75.0 and 77.5, indicating a positive response among experienced patients. In the formal test, the lowest scores came from one therapist who raised several concerns. The therapist specifically suggested that the wrist should remain static during operation and indicated that a patient with Brunnstrom stages 1 or 2 would have trouble griping the handle.

### Weaknesses

During the design process, we were unable to find an adjustable-height table that was able to adequately fulfill the users’ needs and thus future work will address this issue. In addition, the ergonomics of the device need to be improved in order to allow it to fit a wider range of potential users. During operation, the two handles tend to rotate, indicating that there is a need for improvements in some details of the machine. We are developing new functionalities that will provide graded resistance levels, allow the user to control exercise duration, allow recording of the patient’s behavioral performance, and bring about the incorporation of game-based interfaces. Finally, this experiment only involved a small number of participants and future studies will need to recruit a larger pool of individuals to be tested in order to evaluate the device’s clinical usability, thus allowing the device to be more fully applied in the field and to maximize its therapeutic value.

## Conclusion

The *MirrorPath* developed in this study is based on the bilateral movement and PNF theories and uses motor-driven motion to apply passive exercise to both of the patient’s arms, thus both maintaining the range of motion of the shoulders and elbows and enhancing the coordination of both arms. During the course of the experiment, we found that this type of design is better suited to patients who lack voluntarily limb movement. The test results indicate that the device has a good degree of usability, and the observation of the users help to raise various issues that need further improvement. Products always require constant re-engineering and refinement in order to improve their practicality.

## Ethics Statement

This study protocol was specifically approved by the Institutional Review Board of Chang Gung Medical Foundation with ID 101-5038A3. The study has been registered in https://ClinicalTrials.gov with registration number NCT02698605.

## Author Contributions

Y-CP, J-LC, AW, and KT designed and conducted the experiment; KT analyzed these data; Y-CP and KT wrote the manuscript.

## Conflict of Interest Statement

The authors declare that the research was conducted in the absence of any commercial or financial relationships that could be construed as a potential conflict of interest.
